# Insufficient neutralization in testing a chlorhexidine-containing ethanol-based hand rub can result in a false positive efficacy assessment

**DOI:** 10.1186/1471-2334-5-48

**Published:** 2005-06-20

**Authors:** Günter Kampf, Marc Shaffer, Corrine Hunte

**Affiliations:** 1 BODE Chemie GmbH & Co., Scientific Affairs, Melanchthonstrasse 27, 22525 Hamburg, Germany; 2 Institut für Hygiene und Umweltmedizin, Ernst-Moritz-Arndt Universität Greifswald, Walther-Rathenau-Str. 49a, 17489 Greifswald, Germany; 3 Clinical Research Laboratories Inc., 371 Hoes Lane, Piscataway NJ 08854, USA

## Abstract

**Background:**

Effective neutralization in testing hand hygiene preparations is considered to be a crucial element to ensure validity of the test results, especially with the difficulty to neutralize chlorhexidine gluconate. Aim of the study was to measure the effect of chemical neutralization under practical test conditions according to EN 1500.

**Methods:**

We have investigated two ethanol-based hand rubs (product A, based on 61% ethanol and 1% chlorhexidine gluconate; product B, based on 85% ethanol). The efficacy of products (application of 3 ml for 30 s) was compared to 2-propanol 60% (v/v) (two 3 ml rubs of 30 s each) on hands artificially contaminated with *Escherichia coli *using a cross-over design with 15 volunteers. Pre-values were obtained by rubbing fingertips for 1 minute in liquid broth. Post-values were determined by sampling immediately after disinfection in liquid broth with and without neutralizers (0.5% lecithin, 4% polysorbate 20).

**Results:**

The neutralizers were found to be effective and non-toxic. Without neutralization in the sampling fluid, the reference disinfection reduced the test bacteria by 3.7 log_10_, product B by 3.3 log_10 _and product A by 4.8 log_10 _(P = 0.001; ANOVA). With neutralization the reference disinfection reduced the test bacteria by 3.5 log_10_, product B by 3.3 log_10 _and product A by 2.7 log_10 _(P = 0.011; ANOVA). In comparison to the reference treatment Product B lead to a lower mean reduction than the reference disinfection but the difference was not significant (P > 0.1; Wilcoxon-Wilcox test). Without neutralizing agents in the sampling fluid, product A yielded a significantly higher reduction of test bacteria (4.8; P = 0.02) as compared to the situation with neutralizing agents (2.7; P = 0.033).

**Conclusion:**

The crucial step of neutralization lies in the sampling fluid itself in order to stop any residual bacteriostatic or bactericidal activity immediately after the application of the preparation, especially with chlorhexidine gluconate-containing preparations. This is particularly important at short application times such as the 30 s.

## Background

After publication of the new CDC guideline on hand hygiene, hospital hygiene practices are currently changing in many countries [1]. For the post-contamination treatment of healthcare workers' hands, it has been suggested to use alcohol-based hand rubs instead of soap and water [1]. This recommendation is based on a superior efficacy and dermal tolerance of the majority of alcohol-based hand rubs [1,2]. The efficacy of hand hygiene preparations is usually tested in suspension tests (e.g. prEN 12054) and under *in vivo *conditions (e.g. EN 1500). Both norms have been used in many studies to determine the efficacy of preparations for hand hygiene and to determine differences among them [3-5]. Neutralization of residual active agents must be achieved immediately after the end of exposure and is done by a 1:10 dilution, and in addition, chemically by supplementing neutralizing agents to the sampling fluids [6]. The rationale for neutralisation is that, especially at short exposure times like 30 s for hygienic hand disinfection, any remaining bacteriostatic or bactericidal activity of the active agents may lead to a false positive efficacy assessment.

Difficulties in neutralization of chlorhexidine gluconate have been reported before. In 1978 it was already shown that detection of surviving *Staphylococcus aureus *can be completely masked by residual chlorhexidine gluconate which inhibits multiplication in standard efficacy tests without valid neutralization [7]. Three years later it was reported for a hand wash preparation based on 4% chlorhexidine in a dilution of 1:10, that a contamination of S. *aureus *can not be recovered in the absence of neutralizing agents which is indicative of bacteriostatic carry-over effects of the antiseptic [8]. A similar observation was made many years later with *Enterococcus *spp. in a similar test design [9]. If a valid system for neutralization of chlorhexidine was used in suspension tests, 4% chlorhexidine revealed only little activity within 5 min against different strains of vancomycin-susceptible and-resistant *Enterococcus *spp. [10]. In 2002, it was shown that even some commonly used combinations of neutralizing agents (e.g. 3.5% Tween, 0.5% lecithin and 0.5% sodium thiosulfate) may not enable recovery of *Escherichia coli *or S. *aureus *if they are exposed to chlorhexidine gluconate (0.5%). This effect was only seen with chlorhexidine gluconate but not with other non-volatile antimicrobial agents such as povidone iodine (10%), benzalkonium chloride (0.5%) or alkyldiaminoethylglcine hydrochloride (0.5%) [11]. These results have raised concerns on the validity of many studies on the in vitro and in vivo efficacy of chlorhexidine gluconate which have been published over the last decades [2].

So far, the influence of effective neutralization on the results of efficacy assessments was only studied in suspension tests. But in tests under practical conditions such as described by EN 1500, its effect has never been addressed. Therefore, we wanted to verify the effect of successful neutralization in a test under practical conditions (*in vivo*) with two different ethanol-based hand rubs, one of them containing 1% chlorhexidine gluconate.

## Methods

### Products and application

The following preparations were used: Iso-propanol (60%, v/v) as the reference alcohol of EN 1500; product A (3M, St. Paul, USA), based on ethanol (61%, w/w) and chlorhexidine gluconate (1%, w/w); and product B (Bode Chemie GmbH & Co., Hamburg, Germany), based on ethanol (85%, w/w). Product A was chosen as an ethanol-based hand rub with a high concentration of chlorhexidine gluconate, product B was chosen as an ethanol-based hand rub with a higher ethanol concentration but without chlorhexidine gluconate. The reference alcohol was applied according to EN 1500: 2 × 3 ml for a total of 60 s which has been shown to have an excellent reproducibility [12,13]. The two ethanol-based products were applied as commonly done in clinical practice: 1 × 3 ml for a total of 30 s.

### Neutralization

One set of experiments was carried out without any neutralizing agents in the sampling fluids but with 0.5% lecithin and 4% polysorbate 20 in the dilution fluid. The other set of experiments under identical test conditions was carried out with the same combination but in the sampling and the dilution fluids. These agents were chosen as they represent commonly used neutralizing agents in testing chemical disinfectants [5,9].

### Evaluation of neutralizing agents

The neutralizing agents were evaluated according to the "standard test methods for evaluation of inactivators of antimicrobial agents", ASTM E 1054-02 [14]. The test consists of four parts:

#### Test organism viability

Butterfields phosphate buffer was inoculated with a volume of test organism to yield 30 – 100 colony-forming units (CFU) per milliliter (ml). The dilution was vortexed. After 1 min and 1 h, the dilutions were vortexed and 1 ml was pipetted into 2 petri dishes. Approximately 15 – 20 ml of trypticase soy agar (TSA) without neutralizers was poured into the petri dishes.

#### Neutralizer effectiveness

A 1:10 dilution of each hand rub was produced by adding 1 gram of a hand rub to 9 ml of Butterfields buffer supplemented with the neutralizing agents. The dilution was vortexed and 1 ml of the dilution was discarded. Within 5 s, 300 – 1000 CFU of the test strain was added to the dilution to yield 30 – 100 CFU/ml. After 1 min and 1 h, the dilutions were vortexed and 1 ml of each was pipetted into two petri dishes. Approximately 15 – 20 ml of TSA with neutralizing agents were poured into the petri dishes.

#### Neutralizer toxicity

A 1:10 dilution was produced by adding 1 ml of Butterfields phosphate buffer to 9 ml of Butterfields buffer supplemented with the neutralizing agents. The dilution was vortexed and 1 ml of the dilution was discarded. Within 5 s, 300 – 1000 CFU of the test strain was added to the dilution to yield 30 – 100 CFU/ml. After 1 min and 1 h, the dilutions were vortexed and 1 ml of each was pipetted into two petri dishes. Approximately 15 – 20 ml of TSA containing neutralizing agents was poured into the petri dishes.

#### Test material control

A 1:10 dilution of each hand rub was produced by adding 1 ml of the challenge organism suspension to 9 g of the hand rub. The dilution was vortexed. After 1 min and 1 h, the dilutions were vortexed and 1 ml of each was pipetted into two petri dishes. Approximately 15 – 20 ml of TSA without neutralizers was poured into the petri dishes.

#### Culture conditions and incubation

The plates were incubated at 36°C for 24 – 48 h. All test were repeated an additional 2 times, for a total of three replicates.

### Efficacy test of hand rubs

Fifteen volunteers participated in each experiment. Hands were washed for 1 minute with soft soap and dried with paper towels. A contamination fluid was prepared with *E. coli *ATCC 11229 by growing the test organism in two test tubes containing 5 ml of tryptic soy broth (TSB) for 18 – 24 h at 36 ± 1°C. These cultures were inoculated in two bottles with 1 l TSB, incubated at 36 ± 1°C for 18 – 24 h and pooled. It contained between 2 × 10^8 ^and 2 × 10^9 ^CFU ml^-1^. The hands of the volunteers were immersed in the contamination fluid up to the mid-metacarpals for 5 s with fingers spread, and then allowed to air dry for 3 min which resembles a contamination of hands after patient care. Fingertips were rubbed for 1 min in a petri dish containing 10 ml of TSB (pre-values). Either 3 ml of a product or 2 × 3 ml of the reference alcohol (2-propanol, 60% v/v) were applied to the hands (cross-over for each volunteer). The rub-in period was 30 s for each product and 60 s for the reference alcohol. Immediately after the rub-in period, fingertips were rubbed again for 1 min in a petri dish containing TSB with and without neutralisers. For both reference and test procedures, the log counts from the left and right hands of each subject were averaged separately for pre-values and post-values. The arithmetic means of all individual log reduction factors (RF) were calculated. A product shall not be significantly less effective in comparison to the reference disinfection.

### Design and statistics

All three agents were tested in individual experiments. Test subjects were treated as paired groups, the same panel of subjects was used for all three experiments (hence paired). This design was justified with the knowledge that hands are artifically contaminated in each experiment and that the artificial contamination leads to highly reproducible pre-values [12,13]. It is therefore very unlikely that the pre-value is influenced by a treatment of hands with a hand rub prior to the artificial contamination. In a first step, means were compared in each group (with and without neutralizing agents in the sampling fluid) in an analysis of variance (ANOVA; SPSS). In a second step, means were analyzed with the Wilcoxon-Wilcox test (Friedman analysis). A significance level of P = 0.1 is set in EN 1500 [6].

## Results

### Evaluation of neutralizing agents

On average, the viability of the test organism after 1 min and 1 h is characterized by a viable count of 88 ± 9 CFU/ml which is equivalent to a mean of 1.95 ± 0.04 log_10 _CFU/ml. Exposure to the neutralized active agents brought about, on average, 88 ± 5 CFU/ml (1.94 ± 0.03 log_10_). There was no significant difference between the two (P = 0.97; two-sided t-test for paired samples). Exposure of the test organism to the neutralizing agents alone resulted in an average of 90 ± 7 CFU/ml (1.94 ± 0.03 log_10_). There was no significant difference between the two (P = 0.66). The combination of 0.5% lecithin and 4% polysorbate 20 was found to be not toxic to the test organism and to effectively neutralize ethanol and chlorhexidine gluconate.

### Efficacy of hand rubs

Without neutralizing agents in the sampling fluid, the reference treatment reduced the artificial contamination of hands by 3.7 log_10_, product A by 4.8 log_10 _and product B by 3.3 log_10 _(Table 1). With neutralizing agents in the sampling fluid, the reference treatment reduced the artificial contamination of hands by 3.5 log_10_, product A by 2.7 log_10 _and product B by 3.3 log_10_. A significant difference was found between the means of all three treatments in the group with neutralizing agents in the sampling fluid (P = 0.001; ANOVA) and in the group without neutralizing agents in the sampling fluid (P = 0.011). In comparison to the reference treatment, product B lead to a lower mean reduction of 3.3 log_10 _(no neutralization in sampling fluids) and 3.3 log_10 _(neutralization in sampling fluids), the differences, however, were not significant (P > 0.1; Wilcoxon-Wilcox test). Without neutralizing agents in the sampling fluids, product A yielded a mean reduction of 4.8 log_10 _which was significantly higher as compared to the reference treatment (P = 0.02). With neutralizing agents the mean reduction amounted to 2.7 log_10 _which was significantly lower as compared to the reference treatment (P = 0.033). The total number of samples without detectable bacteria after treatment with product A was 9 out of 15 when tested without neutralizing agents in the sampling fluid, and 0 out of 15 with neutralizing agents (Table 1).

**Table 1:** Mean reduction ± SD of *Escherichia coli *from artificially contaminated hands of 15 volunteers by reference treatment (2 × 3 ml of 60% iso-propanol for 60 s) or treatment with product A or product B (each 3 ml for 30 s); experiments differed only by the presence of valid neutralizers in the sampling fluid.

Preparation	Active agent(s) and concentration(s)	No neutralizers in sampling fluid		P-value (vs. reference)*	Neutralizers in sampling fluid		P-value (vs. reference)*
					
		Samples without detectable test bacteria	Mean log_10_-reduction ± SD		Samples without detectable test bacteria	Mean log_10_-reduction ± SD	
Product A	Ethanol (61%, w/w) chlorhexidine gluconate (1%, w/w)	9 / 15	4.8 ± 1.5	0.02	0 / 15	2.7 ± 0.4	0.033
Reference alcohol	Iso-propanol (60%, v/v)	0 / 15	3.7 ± 0.8	n.a.	0 / 15	3.5 ± 0.9	n.a.
Product B	Ethanol (85%, w/w)	0 / 15	3.3 ± 0.7	0.44	0 / 15	3.3 ± 0.7	0.11

## Discussion

For the first time we were able to show that lack of effective neutralizing agents in the sampling fluids leads to false positive efficacy assessments of an ethanol-based hand rub containing 1% chlorhexidine. This effect is explained by residual bacteriostatic or bactericidal activity of chlorhexidine gluconate because the other hand rub based on 85% ethanol without chlorhexidine gluconate was effectively neutralized by a 1:10 dilution alone. In addition, the experiments without neutralization in the sampling fluid showed that in 9 out of 15 samples with product A there was no bacterial growth pointing to residual bacteriostatic or bactericidal activity on the agar plates. With neutralizers in the sampling fluids, however, all 15 samples per experiment revealed countable numbers of colonies. With the reference alcohol alone and with product B not containing chlorhexidine gluconate, countable numbers were found in all 15 experiments, both with and without neutralizing agents in the sampling fluid.

Only few studies with alcohol-based hand rubs containing chlorhexidine gluconate have been carried out with valid neutralization of the active agents. The efficacy of product A for surgical hand disinfection was recently evaluated according to prEN 12791. It was found to be significantly less effective compared with the reference treatment, both in the immediate and the sustained effect [15]. It was ensured that neutralization was effective according to the European test method [15]. Even after 3 hours, product A containing 1% chlorhexidine gluconate did not match with the efficacy of the reference alcohol n-propanol (60%, v/v) alone, indicating that 1% chlorhexidine did not really contribute to the overall efficacy. Another study was recently finished using the same test design and assuring effective neutralization. One preparation contained 70% iso-propanol and 0.5% chlorhexidine gluconate. It was less effective as compared to the reference alcohol n-propanol (60%, v/v) indicating once again that 0.5% chlorhexidine did not really contribute to the overall efficacy (Rotter ML et al. 2005; unpublished data). And in 1990, it was shown that any residual activity of chlorhexidine gluconate on hands can be destroyed simply by using an anionic soap which apparently neutralizes residual chlorhexidine gluconate [16]. These results further support doubts on the real benefit of chlorhexidine gluconate as a non-volatile active agent in hand hygiene.

Neutralization must be effective in order to stop any residual bacteriostatic or bactericidal activity and at the same time it must be non-toxic to the test organism. An ASTM standard [14] and a European norm [17] have been published considering both elements. The test system of the European norm prEN 12054 has been shown previously to be suitable to determine the effectiveness and non-toxicity for standard neutralizing agents [5,18]. We are not aware of any comparative studies for the European and US test methods to determine if one of the two methods may be superior to the other. Both methods include relevant test criteria and may be used for the assessment of effectiveness and non-toxicity for neutralizing agents.

## Conclusion

Lack of effective neutralization may yield false positive efficacy data for alcohol-based hand rubs containing chlorhexidine. The crucial step of neutralization seems to occur in the sampling fluid itself where any residual bacteriostatic or bactericidal activity should be stopped immediately after the preset application time. This is particularly important at short application times such as 30 s.

## Competing interests

The first author is paid employee of Bode Chemie GmbH & Co., Hamburg, Germany.

## Authors' contributions

GK designed the study, analysed the data and wrote the manuscript. MS and CH performed the experiments and participated in writing the manuscript. All authors read and approved the final manuscript.

## Pre-publication history

The pre-publication history for this paper can be accessed here:


